# Laser Machining and In Vitro Assessment of Wollastonite-Tricalcium Phosphate Eutectic Glasses and Glass-Ceramics

**DOI:** 10.3390/ma11010125

**Published:** 2018-01-13

**Authors:** Daniel Sola, Lorena Grima

**Affiliations:** 1 Laboratorio de Óptica, Centro de Investigación en Óptica y Nanofísica, Universidad de Murcia, Campus Espinardo, 30100 Murcia, Spain; 2 Instituto de Ciencia de Materiales de Aragón, Dpto. Ciencia y Tecnología de Materiales y Fluidos, Universidad de Zaragoza-CSIC, 50018 Zaragoza, Spain; lgrima@unizar.es

**Keywords:** laser machining, eutectic glass, eutectic glass-ceramic, bioactive materials, hardness

## Abstract

Bioactivity and ingrowth of ceramic implants is commonly enhanced by a suitable interconnected porous network. In this work, the laser machining of CaSiO_3_‒Ca_3_(PO_4_)_2_ biocompatible eutectic glass-ceramics and glasses was studied. For this purpose, 300 µm diameter craters were machined by using pulsed laser radiation at 532 nm with a pulsewidth in the nanosecond range. Machined samples were soaked in simulated body fluid for 2 months to assess the formation of a hydroxyapatite layer on the surface of the laser machined areas. The samples were manufactured by the laser floating zone technique using a CO_2_ laser. Morphology, composition and microstructure of the machined samples were described by Field Emission Scanning Electron Microscopy, Energy Dispersive X-ray Spectroscopy and micro-Raman Spectroscopy.

## 1. Introduction

Bone tissue engineering for developing 3D scaffolds to regenerate and repair injured tissues is a research field of great interest at present. Among the large variety of materials studied for scaffold manufacturing, the most noteworthy are bioactive glasses discovered by Hench et al. [1]. Moreover, both silicon phosphate and silicon calcium phosphate compounds are particularly interesting because of their bioactivity, defined as the ability of the material to develop a hydroxyapatite layer on its surface to bond to bone tissue [2]. Although the optimal pore size in scaffolds is still the subject of ongoing research and may range between 100 µm and 800 µm, an interconnected porous network that enhances cell proliferation, nutrient delivery, bone ingrowth and vascularisation, without causing an important detriment of its structural stability and mechanical properties, must be considered for a suitable design [3,4,5,6,7,8,9,10].

In particular, Ca_3_(PO_4_)_2_/CaSiO_3_ eutectic composites may allow the development of in situ interconnected pore networks due to the fact that tricalcium phosphate (TCP), Ca_3_(PO_4_)_2_, is resorbable and wollastonite (W), CaSiO_3_, is bioactive [11,12,13]. Since the development of Bioeutectic^®^ ceramic by De Aza et al. in 1997 [11], bioactivity and biocompatibility, both in vitro and in vivo, as well as the optical properties of this binary eutectic composite, have been widely studied [14,15,16,17,18,19,20,21,22,23,24,25].

In this work, the interaction between pulsed laser radiation at 532 nm in the nanosecond range and both W-TCP eutectic glass and glass-ceramic samples has been studied as a first approach to generating a macroporous interconnected structure with controlled dimensions. For this purpose, craters have been machined on the surface of both samples to study the geometrical dimensions and ablation yields as a function of the reference position. In addition, the behaviour of these machined samples after a 2-month period in simulated body fluid, and the characteristics of the hydroxyapatite layer generated after this soaking period have been assessed and characterized by Field Emission Scanning Electron Microscopy (FESEM), Energy Dispersive X-ray Spectroscopy (EDX) and micro-Raman Spectroscopy.

## 2. Experimental

### 2.1. Sample Fabrication

Precursor rods were obtained from the powder mixture of synthetized tricalcium phosphate, Ca_3_(PO_4_)_2_ (purity > 99.9% and 1.50 Ca/P molar ratio) and wollastonite, CaSiO_3_, (high-purity reagent-grade CaSiO_3_, 99 wt. %, −200 mesh, Aldrich Chemistry, Saint Louis, MO, USA) in the eutectic composition, 20% and 80% mol % respectively. The resulting powders were isostatically pressed at 200 MPa for 2 min and the obtained ceramic rods were sintered at 1200 °C for 10 h. Next, a CO_2_ Laser Floating Zone (LFZ) system was used to manufacture W-TCP eutectic glass and glass-ceramic samples and after the growth process samples were annealed at 923° Kelvin for 5 h to relieve the stresses formed during the fast cooling of the solidification front. A more detailed description of this manufacturing process is summarized elsewhere [26,27]. The main advantages of this technique when compared to other directional solidification techniques such as Bridgman and Czochralski are the few amount of material required to explore new composites without the requirement of a crucible, hence avoiding the possible contaminants coming from the melt container, the possibility of growing materials with very high melting points, and both the high radial and axial thermal gradients achieved in the solidification front, up to 10^6^ K/m, which allow controlling the resulting microstructure and hence to manufacture crystals, high melting point composite ceramics and even glasses. Finally, as the growth takes place in a sealed chamber, it is possible to work in different atmospheres [28,29,30]. In particular, the W-TCP eutectic glass and glass-ceramic samples were grown in air at 1000 and 50 mm/h respectively. Finally, prior to the laser machining process, the surface of the samples was flattened and polished using sandpapers and diamond polishing abrasives.

### 2.2. Laser Machining

As the laser machining system, a Q-switched Nd:YVO_4_ laser (PowerLine S3 SHG, Rofin, Bergkirchen, Germany) emitting 5.5 ns pulses at 532 nm with a repetition rate of 15 kHz was used. The laser system was provided with a galvanometric scanning head, controlled by CAD software to deflect the laser beam, and was equipped with a 100 mm optical lens at the output to focus the laser beam. The diameter of the laser beam before the optical lens was 5 mm. The samples were machined with pulse energy and peak power of 68 µJ and 12.36 kW, respectively. Under these conditions, 300 µm diameter craters were machined in both the glass-ceramic and glass samples, deflecting the laser beam in a zigzag movement with a linear scanning speed of 100 mm/s and a lateral distance of 10 µm. To determine the ablation yields and the optimal conditions for the material removal, the samples were processed around the focal plane moving the samples upwards and downwards up to 3 mm, iterating the machining process 20, 40 and 60 times.

### 2.3. In Vitro Tests

After the laser machining process, the samples were immersed in simulated body fluid (SBF) for 2 months aiming at developing a hydroxyapatite (HA) layer on the surface of the samples. The preparation of the simulated body fluid (SBF) was carried out according to the standard process [31], Table 1. During the soaking period, samples were kept at human body temperature of 37 °C by using a Memmert Beschickung stove (Memmert GmbH, model 100–800, Schwabach, Germany).

### 2.4. Characterization Techniques

Surface topography and profile measurements were acquired with an optical confocal microscope (Nikon Sensofar Plμ2300, Terrassa, Spain). Composition and morphology of the processed samples were determined by Field Emission Scanning Electron Microscopy (FESEM) using a Carl Zeiss MERLIN microscope equipped with Energy Dispersive X-ray detector (EDX) (Carl Zeiss microscopy GmbH, Munich, Germany). Structural characterization of the HA layer developed on the surface of the samples was performed by micro-Raman spectroscopy using a confocal Raman Spectrometer (Alpha 300M+, Witec, Ulm, Germany) equipped with a thermoelectric-cooled CCD detector. Samples were excited with a 488 nm laser system and focused on the samples through a 50× microscope objective lens, setting the output power of the laser source at 1 mW. 

## 3. Results and Discussion

**Figure 1:**
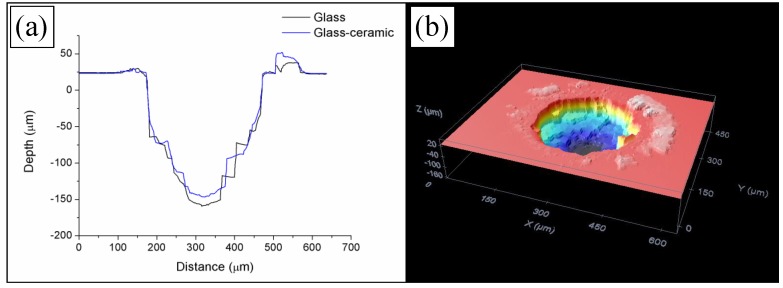
Profile of the machined depth obtained in a 300 µm diameter laser ablated crater at a reference position of −2 mm out of focus (**a**), and topography of the surface of the machined area (**b**).

**Figure 2:**
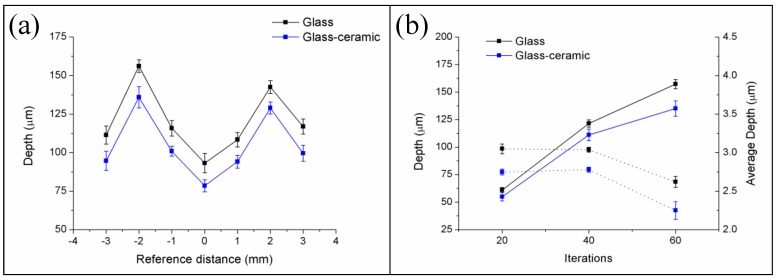
Machined depth achieved for the W-TCP eutectic glass and glass-ceramic samples around the focal plane (cero position) for 60 iterations (**a**) and machined depth (solid lines) and mean average depth (dotted lines) as a function of the number of iterations (**b**).

In the first place, W-TCP eutectic glass and glass-ceramic samples were machined to assess the interaction between the laser radiation at 532 nm in the nanosecond range and the samples, and to determine the ablation rates. For this purpose, samples were processed around the focal plane moving the surface of the samples upwards and downwards up to 3 mm out of focus. The sign convention taken was as follows: negative when the sample was moved upwards, i.e., the focal plane was placed below the surface, and positive when it was moved downwards, i.e., the focal plane was above the surface. To study the machining efficiency and ablation yields, the machining process was carried out by processing the samples for 20, 40 and 60 iterations with the experimental conditions mentioned in Section 2.2. As an example, Figure 1 shows the profiles of the machined depth achieved in a 300 µm diameter crater for both the glass and the glass-ceramic samples for a reference position of −2 mm after iterating the laser processing 60 times, Figure 1a, and a topography of the machined surface, Figure 1b. For these conditions, the maximal depths attained were 157.25 ± 4.21 µm and 134.99 ± 6.93 µm for the glass and the glass-ceramic, respectively. The profiles showed that, due to the brittle nature of the samples, the material was not removed homogeneously, giving rise to abrupt changes of depth. In addition, the topography revealed that part of the ejected material was deposited on the surface. Figure 2a shows the machined depth achieved around the focal plane for both samples and for the case of 60 iterations, and Figure 2b the machined depth achieved (solid lines) and the average depth (dotted lines) as a function of the number of iterations. In Figure 2a it can be observed that the maximal machined depth was achieved by placing the sample out of focus, specifically at −2 mm. As previously reported, the machined depth and the removed volume depend on the laser machining method [28,32,33,34]. When the samples were machined by using the percussion technique—i.e., delivering the whole laser pulses over the same area—the maximal machined depth and removed volume were achieved when the surface of the sample was placed at the focal plane. However, for machining large areas, it is necessary to deflect the laser beam over the sample or to move the sample by using a translation stage if the laser system is not equipped with a scanning head. It was shown that, in this machining method, the maximal machined depth and removed volume were achieved by placing the sample out of focus [32,33,34]. As shown in Figure 2a, this phenomenon also holds for the case of these eutectic glasses and glass-ceramic samples. It is worth mentioning that the machined depth for the glass-ceramic was slightly lower than for the glass, which is related to the higher hardness of the glass-ceramic, 495 HV, versus 458 HV for the glass. It was also demonstrated that there exists a close relation between the ablation yields and the material hardness, such that the harder the material is, the lower the ablation yields [32,34]. Figure 2b depicts that, although the machined depth increased with the number of iterations, the average depth decreased for a number of iterations higher than 40. In particular, for the glass sample, it decreased from around 3 µm for 20 and 40 iterations to 2.62 µm for 60 iterations, and for the glass-ceramic sample, from around 2.75 µm to 2.25 µm. Therefore, the surface to be machined had to be readjusted after 40 iterations for an optimal machining process. For these conditions, the removed volume and the ablation yield were found to be 200 µm^3^/pulse and 340 J/mm^3^ for the glass and 183 µm^3^/pulse and 371 J/mm^3^ for the glass-ceramic sample. These ablation yields might be increased by using ultrashort laser pulses. During the ablation process in the nanosecond range, a plasma is created in the interaction zone. This plasma can shield the surface to be machined, hindering the ablation process [35].

**Figure 3:**
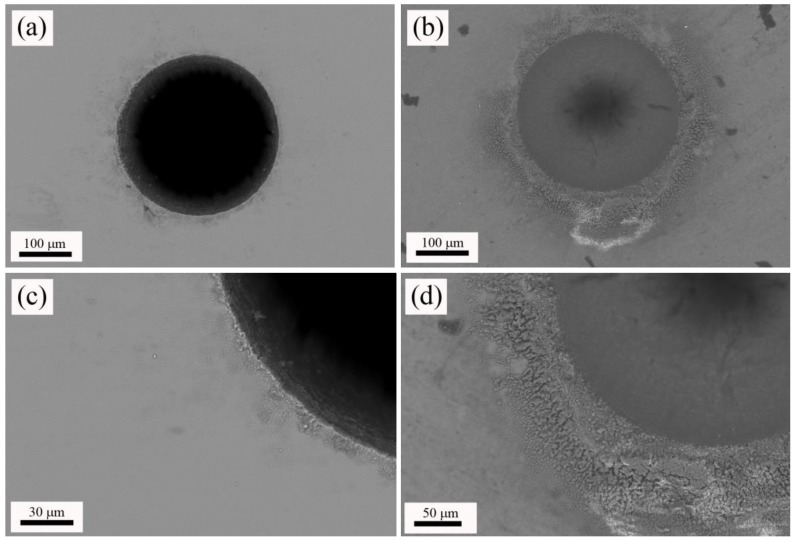
Top-view micrographs of the machined glass (**a**) and glass-ceramic (**b**); and detail of the heat affected zone, HAZ, produced in the surroundings of the processed area in the glass (**c**) and glass-ceramic (**d**).

Next, W-TCP eutectic glass and glass-ceramic machined samples were characterized by means of SEM-EDX. Figure 3a,b shows top-view micrographs of the craters machined in the glass and the glass-ceramic sample, respectively. In both cases, the surroundings of the machined areas presented a heat-affected zone, HAZ, and deposition of the ejected material. The origin of the HAZ relies on the photothermal nature of the interaction between nanosecond laser pulses with matter [36]. Both Figure 3c,d shows these areas in detail. It is worth noting that the glass-ceramic sample presented a higher amount of deposited material than the glass sample. EDX analyses were carried out on both machined samples to evaluate whether the composition of the departing sample was modified. Table 2 shows that composition of both glass and glass-ceramic samples was close to the theoretical eutectic composition. Additionally, these compositional analyses indicated that the HAZ’s compositions were approximately the same as the departing materials.

**Figure 4:**
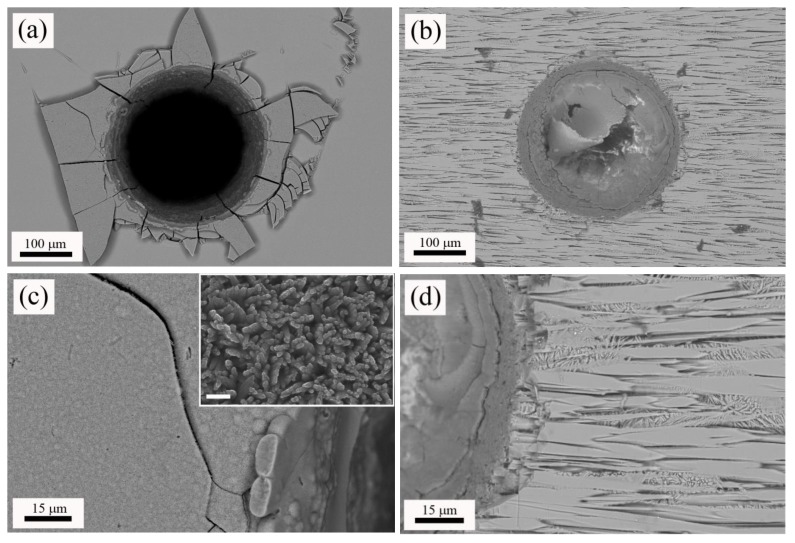
Top-view micrographs of the machined glass (**a**) and glass-ceramic (**b**) after 2 months immersed in SBF, and details of the microstructure in the surroundings of the processed area in the glass (**c**) and glass-ceramic (**d**). The scale bar in the inset of micrograph (**c**) is equal to 200 nm.

**Figure 5:**
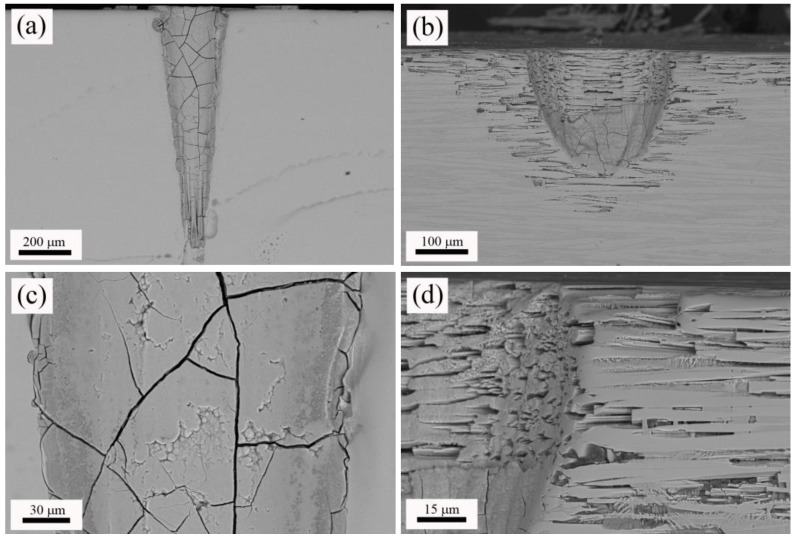
Cross-section micrographs of the machined glass (**a**) and glass-ceramic (**b**) after 2 months immersed in SBF, and details of the microstructure of the processed area in the glass (**c**) and glass-ceramic (**d**).

Next, machined samples were immersed in SBF for 2 months to assess their bioactivity. Figure 4 shows top-view micrographs of the craters machined in the W-TCP eutectic glass and glass-ceramic after this soaking period (a) and (b) respectively, and details of the microstructure found in the surroundings of the machined area for the glass (c) and the glass-ceramic sample (d). Figure 5 shows cross-section micrographs of the craters machined in the W-TCP glass (a) and glass-ceramic (b), and details of the microstructure of the processed area in the glass (c) and glass-ceramic (d). In the glass sample, a new layer was developed on the surface of the sample, Figure 4a, and also on the walls of the machined hole, Figure 5a. This new layer was made up of fibrillar nanocrystals, randomly oriented, as shown in the inset of Figure 4c, hence providing porosity to the layer. This new layer showed cracks not present in the starting glass, which were originated by the difference in the mechanical properties between the layer and the glass. The composition of this layer was characterized by EDX as on the surface as on the wall of the crater revealing that the layer did not contain Si and was rich in P and Ca, with Ca/P ratios of about 1.4 and 1.35 for the layer on the surface and the wall respectively, Table 3.

With regard to the glass-ceramic sample, the microstructure was made up of fibres aligned to the growth direction embedded in matrix, as shown in Figure 4b or Figure 4d, and Figure 5b or Figure 5d. A thorough explanation of the microstructure can be found in previous works [21,22]. In summary, the fibres (clear contrast) contained Si, P and Ca, whereas the matrix (dark contrast) was rich in Si and Ca, with low content of P. EDX analyses carried out in this sample confirmed these results, Table 3. In the glass-ceramic sample, after the soaking period in SBF, the new layer was found only in the lower part of the walls of the machined hole, as shown in Figure 4b or Figure 5b. The composition of this layer was similar to that found in the glass sample, Table 3, with a Ca/P ratio of 1.5. It is highly remarkable that the immersion of the glass-ceramic sample in SBF gave rise to the dissolution of the matrix, rich in Si, whereas the fibres remained unaltered, Figure 4d or Figure 5d and Table 3. Likewise, it is also worth mentioning that although this glass-ceramic was obtained departing from a non-equilibrium state, the mechanisms of dissolution of the Si-rich phase proposed by de Aza et al. still seem to be applicable [14]. Thus, the machining of the craters enhanced the sample becoming a porous 3D structure. 

**Figure 6:**
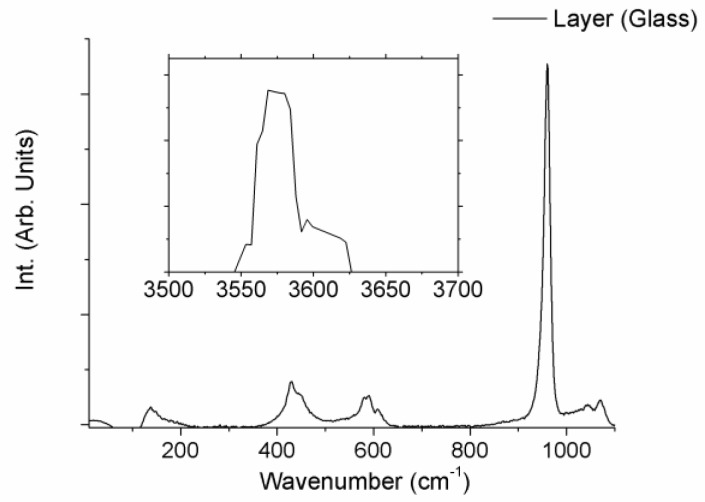
µ-Raman spectra of the layer developed on the surface of the W-TCP eutectic glass sample after 2-month immersed in SBF.

Finally, the structure of the layers produced on the surface of the samples after the immersion period in SBF was evaluated by micro-Raman spectroscopy. Both layers showed the same Raman spectra. As an example, Figure 6 shows the Raman spectra of the layer developed on the glass sample, for both 50–1200 cm^−1^, and 3400–3800 cm^−1^ wavenumber regions. Raman spectra showed broad bands placed at 400–500, 570–625 and 1020–1095 cm^−1^, a narrow intense peak located at 962 cm^−1^ and a strong sharp peak at 3576 cm^−1^. These Raman features can undoubtedly be identified to the Raman spectra of hydroxyapatite, HA [11,18]. Hence, micro-Raman characterization verified the HA nature of the layer developed on both samples during the soaking period.

## 4. Conclusions

The interaction between pulsed laser radiation in the nanosecond range and W-TCP eutectic glass and glass-ceramic samples manufactured by the Laser Floating Zone technique was assessed as a first approach for generating 3D interconnected porous structures. Ablation yields obtained for both samples showed that the optimal machining conditions were achieved by placing the sample out of focus. In addition, the relation between ablation yields and material hardness was confirmed, such that the harder material, the glass-ceramic, gave rise to a lower ablation yield.

Both W-TCP eutectic glass and glass-ceramic machined samples were immersed in simulated body fluid for 2 months to assess their bioactivity. In the glass sample, a hydroxyapatite layer was developed on the surface of the wholes sample, walls of machined crater included, with a Ca/P ratio around 1.35 and 1.4. Concerning the glass-ceramic sample, the hydroxyapatite layer was found only in the surface of the walls of the machined hole, with a Ca/P ratio of 1.5. Furthermore, the soaking in SBF produced the dissolution of the Si-rich matrix, giving rise to a porous fibrillar structure so that the laser machined holes enhanced the sample becoming a 3D porous structure. Micro-Raman characterization confirmed that the layers developed on both samples corresponded to hydroxyapatite. 

## Figures and Tables

**Table 1:** Composition of the simulated body fluid, in mM, prepared for the in vitro tests.

Na^+^	K^+^	Mg^2+^	Ca^2+^	Cl^−^	HCO_3_^−^	HPO_4_^2−^	SO_4_^2−^
142.0	5.0	1.5	2.5	147.8	4.2	1.0	0.5

**Table 2:** EDX compositional analysis in at % of both W-TCP glass and glass-ceramic sample.

Sample	Si	P	Ca
Glass (G)	11.00	6.48	18.96
Glass-ceramic (GC)	10.01	6.40	16.89
HAZ (G)	10.89	6.42	18.07
HAZ(GC)	10.71	6.33	16.44

**Table 3:** EDX compositional analysis in at % of the microstructure found in the W-TCP machined glass and glass-ceramic samples.

Sample	Stage	Site of Interest	Si	P	Ca
Glass	Soaked	Layer, Surface	–	13.92	19.48
Glass	Soaked	Layer, Wall	–	12.88	17.45
Glass-ceramic	Non-soaked	Fibre (Clear contrast)	5.82	9.74	19.50
Glass-ceramic	Non-soaked	Matrix (Dark contrast)	18.20	0.47	15.70
Glass-ceramic	Soaked	Layer, Wall	–	12.57	19.08
Glass-ceramic	Soaked	Fibre (Clear contrast)	6.10	9.91	20.45
